# Relationship between lower urinary system symptoms and urinary incontinence in women with gestational diabetes

**DOI:** 10.1590/1806-9282.20251467

**Published:** 2026-06-26

**Authors:** Leyla Kaya, Esra Keles, Zahide Kaya, Hakan Dokumuş

**Affiliations:** 1 University of Health Sciences, Faculty of Health Sciences, Department of Midwifery – Istanbul, Turkey.; 2 University of Health Sciences, Kartal Lütfi Kırdar City Hospital, Department of Gynecologic Oncology – Istanbul, Turkey.; 3 University of Health Sciences, Hamidiye Faculty of Medicine, Department of Public Health – Istanbul, Turkey.; 4 Uskudar State Hospital, Department of Internal Medicine – Istanbul, Turkey.; 5 Mersin University, Institute of Health Sciences, Department of Nursing – Mersin, Turkey.

**Keywords:** Gestational diabetes mellitus, Urinary incontinence, Quality of life, Pregnancy, Lower urinary tract symptoms, Pelvic floor disorders

## Abstract

**OBJECTIVE:**

The aim of this study was to evaluate the association between gestational diabetes mellitus and the severity and impact of lower urinary tract symptoms and urinary incontinence on quality of life in pregnant women.

**METHODS:**

This case–control study enrolled 500 pregnant women (251 with gestational diabetes mellitus; 249 healthy controls) between 30 and 38 weeks’ gestation. Women completed the International Consultation on Incontinence Questionnaire-Short Form, Urogenital Distress Inventory-Short Form, Incontinence Impact Questionnaire-Short Form, and Incontinence Severity Index. Sociodemographic, obstetrics, and anthropometric data were also collected. Multivariable logistic regression analyses were performed to examine the independent association between gestational diabetes mellitus and lower urinary tract symptoms and urinary incontinence.

**RESULTS:**

The gestational diabetes mellitus group exhibited a significantly higher mean age compared to the control group (p<0.001). Pre-pregnancy body mass index and body mass index during pregnancy were also significantly elevated in the gestational diabetes mellitus group (p<0.001). The median gestational weight gain was significantly greater in the gestational diabetes mellitus group (p=0.027). Women with gestational diabetes mellitus demonstrated significantly elevated scores in International Consultation on Incontinence Questionnaire-Short Form, Urogenital Distress Inventory-6, Incontinence Impact Questionnaire-7, and Incontinence Severity Index (p<0.001 for all). After adjusting for age, pre-pregnancy body mass index, body mass index during pregnancy, and gestational weight gain in multivariable logistic regression models, gestational diabetes mellitus remained independently associated with increased UI risk (adjusted OR 2.34, 95%CI 1.56–3.51, p<0.001).

**CONCLUSION:**

Gestational diabetes mellitus is significantly associated with increased severity of lower urinary tract symptoms and urinary incontinence, as well as a greater negative impact on quality of life during pregnancy. Routine systematic screening for lower urinary tract symptoms and urinary incontinence may be considered for all pregnant women with gestational diabetes mellitus, with early implementation of preventive interventions including supervised pelvic floor muscle training programs.

## INTRODUCTION

Gestational diabetes mellitus (GDM), characterized by the onset or initial diagnosis of glucose dysregulation during pregnancy, represents the most prevalent metabolic complication impacting approximately 1–14% of pregnancies globally, with prevalence rates increasing in parallel with rising trends in obesity and advanced maternal age^1^. GDM confers significant short-term maternal and fetal risks, including macrosomia, preeclampsia, polyhydramnios, and neonatal hypoglycemia. Furthermore, it elevates long-term maternal susceptibility to Type 2 diabetes mellitus (T2DM) and cardiovascular disease^2^.

Concurrently, lower urinary tract symptoms (LUTS), encompassing a range of bothersome conditions including urinary incontinence (UI), urgency, frequency, and nocturia, are exceedingly common during pregnancy^3^. The physiological adaptations of pregnancy, such as increased intra-abdominal pressure exerted by the gravid uterus on the bladder and pelvic floor, hormonal changes affecting connective tissue and muscle tone, and potential neurogenic alterations, contribute significantly to the development or exacerbation of these symptoms^4^.

Existing literature has established a clear association between T2DM and heightened UI risk in females^5^. The proposed mechanisms include diabetic neuropathy affecting bladder innervation and sphincter function, diabetic myopathy impacting pelvic floor muscle (PFM) strength, and microvascular complications affecting pelvic tissue integrity^6^. Given the metabolic similarities and shared risk factors (such as obesity and advanced age) between GDM and T2DM, it is plausible that GDM may also predispose pregnant women to LUTS/UI or exacerbate existing symptoms^7^. However, the direct link between GDM and UI during the index pregnancy is less definitively established compared to the long-term postpartum period^8^. Reported prevalence rates of LUTS/UI in pregnant women with GDM range from 35 to 68%, depending on diagnostic criteria, population characteristics, and assessment instruments employed^9,10,11^. Cross-cultural studies suggest that while the GDM–LUTS/UI association appears consistent across populations, the magnitude of effect may be influenced by population-specific factors including ethnicity, healthcare access, and cultural attitudes toward symptom reporting.

Given the significant prevalence of both GDM and LUTS in pregnancy, and the potential impact on maternal well-being, a comprehensive understanding of the association between these two conditions is crucial. This study aimed to investigate the severity and impact on quality of life (QoL) of LUTS and UI in pregnant women with GDM compared to those with uncomplicated pregnancies, utilizing a battery of validated questionnaires to provide a quantitative assessment.

## METHODS

This case–control study was conducted at Zeynep Kamil Women’s and Children’s Disease Research and Training Hospital, between April 2023 and April 2024. Pregnant women diagnosed with GDM (case group) were prospectively enrolled and compared to a healthy gestational age-matched control group receiving routine antenatal care during the same period.

Inclusion criteria for both groups included singleton pregnancy, ability to understand and complete the study questionnaires, and gestational age between 30 and 40 weeks. Exclusion criteria comprised pre-existing diabetes mellitus (Type 1 or 2), history of prior urinary tract surgery or known urogenital anomalies, current urinary tract infection (confirmed by urine analysis/culture), known neurological conditions affecting bladder function, multiple pregnancies, history of psychiatric illness, use of medications known to affect bladder function, and refusal to participation to study.

### Data collection and definitions

GDM was diagnosed according to either the two-step approach (American College of Obstetricians and Gynecologists [ACOG] guidelines: 50-g glucose challenge test followed by 100-g oral glucose tolerance test [OGTT] for screen-positive individuals) or the one-step criteria (International Association of the Diabetes and Pregnancy Study Groups [IADPSG]: 50-g OGTT)^9,10^.

Sociodemographic information included age, parity, educational level, occupation, income, smoking, alcohol consumption, coffee/tea consumption, previous mode of delivery, and daytime/nighttime urination frequency. Gestational weight gain was calculated as the difference between current weight and pre-pregnancy weight.

### Urogenital Distress Inventory-6

This 6-item instrument measures distress from irritative (items 1–2), stress (items 3–4), and obstructive/voiding symptoms (items 5–6) on a 4-point Likert scale (0=“not at all” to 3=“greatly”). Higher total scores indicate greater distress^11^. The Turkish version was validated by Çam and Sakallı, showing strong reliability (Cronbach’s α=0.74–0.87)^12^.

### Incontinence Impact Questionnaire-7

This 7-item scale evaluates UI’s impact on physical activity, social relationships, travel, and emotional well-being using a 4-point Likert scale. Scores are standardized (0–100); higher scores denote greater impact. The Turkish version demonstrated excellent internal consistency (Cronbach’s α=0.87) and test– retest reliability (Spearman’s ρ=0.99)^12^.

### International Consultation on Incontinence Questionnaire-Short Form

This instrument assesses the severity and impact of UI over the past 4 weeks. The total score is derived from three core items evaluating frequency, volume of leakage, and the impact on QoL (items 3–5), yielding a 0–21 severity score, with higher scores indicating worse symptoms. Items 1 and 2 are demographic, while item 6 assesses the perceived cause of leakage and is not scored. The Turkish version of the ICIQ-SF has been validated by Cetinel et al^13^.

### Sandvik Severity Index (Incontinence Severity Index)

This index quantifies UI severity by multiplying the frequency of leakage episodes by the amount of leakage. Based on the resulting score, UI is classified as mild (1–2), moderate (3–6), or severe (8–12). The ISI has been widely used in clinical research to stratify incontinence severity^14^.

### Statistical analysis

Analyses were performed using IBM^®^ SPSS^®^ Statistics 30. Continuous variables are summarized as mean±standard deviation or median (interquartile range) based on normality assessed via Kolmogorov-Smirnov test, skewness, and kurtosis. Categorical variables are presented as frequencies (percentages). Non-parametric tests were employed due to non-normal distributions. Group comparisons (GDM vs. control) for UDI-6, ICIQ-SF, IIQ-7, and Sandvik Severity Index scores used the Mann-Whitney U test (effect size: r=Z/√N; thresholds: 0.10=small, 0.30=medium, and 0.50=large). Categorical variable homogeneity was assessed using the chi-square test. Relationships between continuous variables were explored via Spearman’s correlation analysis. Multivariable logistic regression was performed to examine the independent association between GDM and UI (defined as ICIQ-SF score ≥1) while adjusting for potential confounding variables including age, pre-pregnancy body mass index (BMI), BMI during pregnancy, and gestational weight gain. Adjusted odds ratios (ORs) with 95%CIs were calculated. Questionnaire internal consistency was evaluated using Cronbach’s alpha. Statistical significance was defined as p<0.05.

## RESULTS

A total of 500 pregnant women participated in the study, with 251 in the GDM group and 249 in the control (non-GDM) group. The sociodemographic characteristics of the participants are presented in Table 1.

**Table 1:** Sociodemographic characteristics of pregnant women with and without gestational diabetes mellitus.

Groups	GDM group (n=251)	Control group (n=249)	Total (n=500)	Test value	p-value
Age (years)	30.96±6.2330 (26–35)	27.96±5.0228 (24–31)	29.47±5.8529 (25–33)	Z=-5.062	**p<0.001**
Cigarettes/day	0.92±3.250 (0–0)	0.43±1.550 (0–0)	0.68±2.560 (0–0)	Z=-1.001	p=0.317
Education level				χ^2^=1.197	p=0.754
Primary school	40 (15.9%)	33 (13.3%)	73 (14.6%)		
Middle school	35 (13.9%)	38 (15.3%)	73 (14.6%)		
High school	127 (50.6%)	123 (49.4%)	250 (50.0%)		
University	49 (19.5%)	55 (22.1%)	104 (20.8%)		
Employment status				χ^2^=0.000	p=1.000
Employed	47 (18.7%)	46 (18.5%)	93 (18.6%)		
Unemployed	204 (81.3%)	203 (81.5%)	407 (81.4%)		
Income status				χ^2^=2.095	p=0.351
Income<expenses	15 (6.0%)	21 (8.4%)	36 (7.2%)		
Income=expenses	203 (80.9%)	203 (81.5%)	406 (81.2%)		
Income>expenses	33 (13.1%)	25 (10.0%)	58 (11.6%)		
Smoking status				χ^2^=0.734	p=0.392
Yes	29 (11.6%)	22 (8.8%)	51 (10.2%)		
No	222 (88.4%)	227 (91.2%)	449 (89.8%)		
Coffee consumption				χ^2^=1.561	p=0.212
0 cups/day	139 (55.4%)	123 (49.4%)	262 (52.4%)		
1–3 cups/day	112 (44.6%)	126 (50.6%)	238 (47.6%)		
Tea consumption				χ^2^=3.993	p=0.136
0 cups/day	34 (13.5%)	29 (11.6%)	63 (12.6%)		
1–3 cups/day	127 (50.6%)	148 (59.4%)	275 (55.0%)		
≥4 cups/day	90 (35.9%)	72 (28.9%)	162 (32.4%)		

Data are presented as mean±standard deviation or median (interquartile range) for continuous variables and number (percentage) for categorical variables. x̄: mean; SD: standard deviation; M (IQR): median (25th–75th percentiles); n: number; %: percent; Z: Mann-Whitney U standardized test statistic; χ^2^: chi-square test; GDM: gestational diabetes mellitus. Statistically significant values are denoted in bold.

The mean age of the participants with GDM was significantly higher than that of the control group (30.96±6.23 vs. 27.96±5.02 years; p<0.001). Pre-pregnancy BMI and BMI during pregnancy were also significantly higher among those with GDM (p<0.001). The median gestational weight gain was significantly greater in the GDM group (15 kg vs. 14 kg; p=0.027). No significant differences were found in terms of smoking, parity, education level, employment status, income level, alcohol consumption, coffee and tea intake, previous mode of delivery, and frequency of nighttime urination (p>0.05) (Tables 1 and 2).

**Table 2:** Distribution of sociodemographic, obstetric, and incontinence-related characteristics of pregnant women by group.

Group	GDM group (n=251)	Control group (n=249)	Total (n=500)	Test value	p-value
Pre-pregnancy BMI (kg/m^2^)	26.20±3.4726 (24–28)	24.39±3.8524 (22–26)	25.30±3.7725 (23–27)	Z=-6.090	**p<0.001**
BMI during pregnancy (kg/m^2^)	31.93±3.6432 (30–34)	29.79±4.0929 (27–32)	30.86±4.0131 (29–33)	Z=-6.917	**p<0.001**
Gestational weight gain (kg)	15.04±5.4715 (12–18)	14.25±4.6314 (11–17)	14.65±5.0814 (11–18)	Z=-2.205	**p=0.027**
Gestational week	38.60±1.6439 (38–40)	38.63±1.8039 (38–40)	38.62±1.7239 (38–40)	Z=-0.807	p=0.420
Number of pregnancies	2.27±1.292 (1–3)	2.23±1.332 (1–3)	2.25±1.312 (1–3)	Z=-0.617	p=0.537
Number of living children	0.98±1.021 (0–2)	0.92±1.071 (0–2)	0.95±1.041 (0–2)	Z=-1.107	p=0.268
Previous birth type				χ^2^=3.167^a^	p=0.205
Vaginal	97 (38.6%)	114 (45.8%)	211 (42.2%)		
Cesarean	59 (23.5%)	46 (18.5%)	105 (21.0%)		
Instrumental (vacuum/kiwi)	95 (37.8%)	89 (35.7%)	184 (36.8%)		
Nighttime urination frequency				χ^2^=6.025^a^	p=0.197
Never	5 (2.0%)	2 (0.8%)	7 (1.4%)		
Once	23 (9.2%)	28 (11.2%)	51 (10.2%)		
Twice	65 (25.9%)	84 (33.7%)	149 (29.8%)		
Three times	78 (31.1%)	68 (27.3%)	146 (29.2%)		
Four times	80 (31.9%)	67 (26.9%)	147 (29.4%)		

Data are presented as mean±standard deviation or median (interquartile range) for continuous variables and number (percentage) for categorical variables. x̄: mean; SD: standard deviation; M (IQR): median (25th–75th percentiles); n: number; %: percent; Z: Mann-Whitney U standardized test statistic; χ^2^: chi-square test; GDM: gestational diabetes mellitus; BMI: body mass index.

^a^Pearson’s chi-square test. Statistically significant values are denoted in bold.

Pregnant women with GDM scored significantly higher across all incontinence and QoL instruments compared to those without GDM (*p*<0.001 for all comparisons, Table 3). The mean ICIQ-SF score was significantly higher in the GDM group (7.16±5.65) than that in the control group (4.55±5.37; *p*<0.001). The UDI-6 scores were also significantly elevated in the GDM group (8.87±4.41 vs. 6.83±4.52; *p*<0.001). The IIQ-7 and ISI scores were higher in the GDM group (7.39±7.13 vs. 4.45±6.34, *p*<0.001; and 2.81±2.97 vs. 1.74±2.73; *p*<0.001, respectively) (Table 3).

**Table 3:** Comparison of Incontinence Severity Index, Urogenital Distress Inventory-6, Incontinence Impact Questionnaire-7, and International Consultation on Incontinence Questionnaire-Short Form scores by group and reliability coefficients

Scale	GDM group (n=251)	Control group (n=249)	Z	p-value	r	Cronbach’s α (total)
ICIQ-SF	7.16±5.65 (0–21)	4.55±5.37 (0–21)	-5.022	<0.001	0.22	0.855
UDI-6	8.87±4.41 (0–18)	6.83±4.52 (0–16)	-5.334	<0.001	0.24	0.793
IIQ-7	7.39±7.13 (0–21)	4.45±6.34 (0–21)	-4.898	<0.001	0.22	0.955
ISI	2.81±2.97 (0–9)	1.74±2.73 (0–9)	-5.838	<0.001	0.26	0.917

M (IQR): median (interquartile range: 25th–75th percentile); x̄: mean; SD: standard deviation; min–max: minimum and maximum values; ☐: Cronbach’s alpha reliability coefficient; Z: standardized Z value from Mann-Whitney U test; r: effect size for Mann-Whitney U test; GDM: gestational diabetes mellitus; ICIQ-SF: International Consultation on Incontinence Questionnaire-Short Form; UDI-6: Urogenital Distress Inventory-6; IIQ-7: Incontinence Impact Questionnaire-7; ISI: Incontinence Severity Index

Multivariable logistic regression analysis was conducted to assess whether GDM remained independently associated with UI after controlling for known confounding variables. In the unadjusted model, GDM was significantly associated with UI (OR 2.87, 95%CI 1.96–4.21, p<0.001). After adjusting for age, pre-pregnancy BMI, BMI during pregnancy, and gestational weight gain, GDM remained independently and significantly associated with increased UI risk (adjusted OR 2.34, 95%CI 1.56–3.51, p<0.001).

## DISCUSSION

This study shows that GDM is associated with significantly higher prevalence and severity of LUTS, including UI, as well as a greater negative impact on incontinence-related QoL among pregnant women. The GDM group scored significantly higher on all validated questionnaires (ICIQ-SF, UDI-6, IIQ-7, and ISI) designed to assess UI frequency, amount, symptom bother (urogenital distress), and impact on daily life. In addition, multivariable regression analysis demonstrated that GDM remained independently associated with increased UI even after adjusting for age, BMI, and gestational weight gain. Our findings demonstrate consistency with large-scale studies from North America, Europe, and Asia that have reported elevated LUTS/UI prevalence in pregnant women with GDM compared to normoglycemic controls^15,16,17,18^.

Several potential pathophysiological mechanisms could explain the observed relationship between GDM and increased LUTS/UI during pregnancy. Chronic hyperglycemia, a hallmark of diabetes and present to varying degrees in GDM, is known to adversely affect various tissues, including muscle, nerve, and connective tissue, all of which are critical for maintaining pelvic floor integrity and function^16^. Diabetic neuropathy could impair the neural control of the bladder detrusor muscle and urethral sphincters, potentially leading to symptoms such as urgency, frequency, and incomplete emptying, as well as sphincter weakness contributing to stress incontinence^6,17^. Diabetic myopathy might weaken the PFMs, which are crucial for urethral support and continence. Furthermore, diabetes can disrupt connective tissue metabolism, affecting the collagen and elastin content and organization in pelvic support structures, potentially leading to fascial and ligamentous laxity^16,18^.

Our multivariable regression analysis specifically addressed the important consideration raised regarding the influence of factors that were more prevalent in the GDM group, including age and BMI. The GDM participants were significantly older and had higher pre-pregnancy and pregnancy BMIs, as well as greater gestational weight gain. These factors are well-established independent risk factors for LUTS and UI during and after pregnancy^19^. Higher BMI and greater weight gain increase intra-abdominal pressure, which can strain the pelvic floor and exacerbate incontinence. However, the persistence of a significant GDM–UI association supports the notion that the metabolic derangements specific to GDM, including chronic hyperglycemia, oxidative stress, and inflammatory cytokine dysregulation, may directly affect pelvic floor tissues through mechanisms distinct from those related to mechanical factors alone^8,15^.

The significantly higher scores on the UDI-6 and IIQ-7 questionnaires in the GDM group are particularly concerning, highlighting the substantial negative impact of LUTS/UI on their QoL. UI and bothersome LUTS can lead to social isolation, avoidance of physical activity, emotional distress, anxiety, depression, and reduced self-confidence during a period that already presents unique physical and psychological challenges^3,20^. Qualitative investigations from various cultural contexts have documented that pregnant women with GDM experience compounded distress when LUTS/UI symptoms are superimposed upon the already-demanding self-management requirements of glycemic control, dietary modification, and frequent monitoring^21,22^. This intersection of metabolic and urogenital symptom burden underscores the imperative for integrated, holistic management approaches.

These findings have significant implications for clinical practice. Routine, systematic screening for LUTS/UI may be considered for all pregnant women with GDM. Brief, validated instruments such as the ICIQ-SF can be efficiently integrated into standard antenatal care. Early identification of symptomatic women facilitates timely, conservative interventions, particularly supervised PFM training, which is effective in reducing pregnancy-associated incontinence^23,24^. Recent randomized trials demonstrate significant continence and QoL improvements with pelvic floor interventions specifically in pregnant women with GDM^16,17,18,19,20^. Pregnant women with GDM and moderate-to-severe LUTS/UI may benefit from referral to urogynecology or pelvic floor physical therapy for comprehensive assessment and individualized treatment. Proactive management of LUTS/UI in pregnant women with GDM may improve immediate QoL and potentially reduce persistent postpartum pelvic floor dysfunction, conferring benefits beyond the pregnancy.

## Limitations

While this study provides strong evidence of the association between GDM and increased LUTS/UI and diminished QoL during pregnancy, it is not without limitations. As a case–control study, our design cannot definitively establish causal relationships or determine the temporal sequence of GDM diagnosis and LUTS/UI onset. Longitudinal studies are needed to track the incidence and progression of LUTS/UI throughout pregnancy and into the postpartum period in women with GDM compared to non-GDM controls. Prospective cohort investigations that assess women prior to GDM diagnosis and follow them longitudinally would provide stronger evidence regarding causality and enable examination of dose–response relationships between glycemic control and symptom severity. Investigating the efficacy of specific PFM training or other conservative management programs specifically in the GDM population is also warranted. Although validated questionnaires are robust tools for assessing subjective symptoms and impact, complementing these with objective measures, such as bladder diaries, pad tests, or urodynamic studies, where clinically indicated, could provide a more complete picture of bladder function. Finally, while we attempted to match groups by gestational age, we acknowledge that the GDM and control groups were not fully homogeneous with respect to age and BMI. Although we addressed this limitation through multivariable regression adjustment, future case–control studies employing individual matching or propensity score matching on these variables would further strengthen causal inference.

## CONCLUSION

This study provides compelling evidence that GDM is significantly associated with increased severity of LUTS/UI. Women with GDM reported higher levels of UI severity, symptom distress, and impact on QoL compared to normoglycemic pregnant women.

## Data Availability

The datasets generated and/or analyzed during the current study are available from the corresponding author upon reasonable request.

## References

[1] Metsärinne K, Pietilä M, Kantola I, Stenman LK, Hätinen OP, Vesikansa A (2022). The majority of type 2 diabetic patients in Finnish primary care are at very high risk of cardiovascular events: a cross-sectional chart review study (STONE HF).. Prim Care Diabetes.

[2] Simmons D, Immanuel J, Hague WM, Teede H, Nolan CJ, Peek MJ (2023). Treatment of gestational diabetes mellitus diagnosed early in pregnancy.. N Engl J Med.

[3] Craig L, Sims R, Glasziou P, Thomas R (2020). Women’s experiences of a diagnosis of gestational diabetes mellitus: a systematic review.. BMC Pregnancy Childbirth.

[4] Tähtinen RM, Cartwright R, Tsui JF, Aaltonen RL, Aoki Y, Cárdenas JL (2016). Long-term impact of mode of delivery on stress urinary incontinence and urgency urinary incontinence: a systematic review and meta-analysis.. Eur Urol.

[5] Løwenstein E, Jepsen R, Andersen LL, Laigaard J, Møller LA, Gaede P (2021). Prevalence of urinary incontinence among women with diabetes in the Lolland-Falster Health Study, Denmark.. Neurourol Urodyn.

[6] Vesentini G, Barbosa AMP, Damasceno DC, Marini G, Piculo F, Matheus SMM (2020). Alterations in the structural characteristics of rectus abdominis muscles caused by diabetes and pregnancy: a comparative study of the rat model and women.. PLoS One.

[7] Tagami K, Iwama N, Hamada H, Tomita H, Kudo R, Kumagai N (2025). Advanced maternal age is a risk factor for both early and late gestational diabetes mellitus: the Japan Environment and Children’s Study.. J Diabetes Investig.

[8] Sartorao CI, Nunes SK, Magyori ABM, Calderon IMP, Barbosa AMP, Rudge MVC (2023). The role of gestational diabetes mellitus and pelvic floor 3D-ultrasound assessment during pregnancy predicting urinary incontinence: a prospective cohort study.. BMC Pregnancy Childbirth.

[9] American Diabetes Association. (2011). Diagnosis and classification of diabetes mellitus.. Diabetes Care.

[10] Metzger BE, Gabbe SG, Persson B, Buchanan TA, Catalano PA, International Association of Diabetes and Pregnancy Study Groups Consensus Panel (2010). International association of diabetes and pregnancy study groups recommendations on the diagnosis and classification of hyperglycemia in pregnancy.. Diabetes Care.

[11] Uebersax JS, Wyman JF, Shumaker SA, McClish DK, Fantl JA (1995). Short forms to assess life quality and symptom distress for urinary incontinence in women: the Incontinence Impact Questionnaire and the Urogenital Distress Inventory. Continence Program for Women Research Group.. Neurourol Urodyn.

[12] Cam C, Sakalli M, Ay P, Cam M, Karateke A (2007). Validation of the short forms of the incontinence impact questionnaire (IIQ-7) and the urogenital distress inventory (UDI-6) in a Turkish population.. Neurourol Urodyn.

[13] Cetinel B, Ozkan B, Can G. (2004). The validation study of ICIQ-SF Turkish version.. Turk Uroloji Dergisi.

[14] Hazar U, Şirin A (2008). İnkontinans şiddet indeksinin geçerlik ve güvenirliği çalışması.. Adnan Menderes Üniv Tıp Fakültesi Dergisi.

[15] Cozier YC, Castro-Webb N, Bertrand KA, Ndama M, Chai TC, Harlow BL (2025). Gestational and Type 2 diabetes in relation to urinary incontinence in black women in the U.S.. Uro.

[16] Wu Y, Li T, Cai F, Ye X, Xu M (2024). Stable pelvic floor muscle training improves urinary incontinence in women with gestational diabetes mellitus.. J Obstet Gynaecol.

[17] França DCH, França EL, Sobrevia L, Barbosa AMP, Honorio-França AC, Rudge MVC (2023). Integration of nutrigenomics, melatonin, serotonin and inflammatory cytokines in the pathophysiology of pregnancy-specific urinary incontinence in women with gestational diabetes mellitus.. Biochim Biophys Acta Mol Basis Dis.

[18] Arslan M, Kozan R (2025). Pelvic floor dysfunction in patients with gestational diabetes mellitus.. World J Diabetes.

[19] Ansarzadeh S, Salehi L, Mahmoodi Z, Mohammadbeigi A (2020). Factors affecting the quality of life in women with gestational diabetes mellitus: a path analysis model.. Health Qual Life Outcomes.

[20] Yavuz A, Kocaöz S, Kara P, Destegül E (2022). The effects of gestational diabetes on lower urinary tract symptoms of pregnant women: a case-control study.. J Obstet Gynaecol.

[21] Henderson J, Alderdice F, Redshaw M (2019). Factors associated with maternal postpartum fatigue: an observationalstudy.. BMJ Open.

[22] Parsons J, Sparrow K, Ismail K, Hunt K, Rogers H, Forbes A (2019). A qualitative study exploring women’s health behaviours after a pregnancy with gestational diabetes to inform the development of a diabetes prevention strategy.. Diabet Med..

[23] Mørkved S, Bø K (2014). Effect of pelvic floor muscle training during pregnancy and after childbirth on prevention and treatment of urinary incontinence: a systematic review.. Br J Sports Med.

[24] Woodley SJ, Boyle R, Cody JD, Mørkved S (2017). Hay-Smith EJC. Pelvic floor muscle training for prevention and treatment of urinary and faecal incontinence in antenatal and postnatal women.. Cochrane Database Syst Rev..

